# Ventilator-derived carbon dioxide production to assess energy expenditure in critically ill patients: proof of concept

**DOI:** 10.1186/s13054-015-1087-2

**Published:** 2015-10-22

**Authors:** Sandra N. Stapel, Harm-Jan S. de Grooth, Hoda Alimohamad, Paul W G Elbers, Armand R J Girbes, Peter J M Weijs, Heleen M. Oudemans-van Straaten

**Affiliations:** Department of Adult Intensive Care Medicine, VU University Medical Center, De Boelelaan 1117, 1181HV Amsterdam, The Netherlands; Research VUmc Intensive Care (REVIVE), VU University Medical Center, De Boelelaan 1117, 1181HV Amsterdam, The Netherlands; Institute of Cardiovascular Research (ICaR-VU), VU University Medical Center, De Boelelaan 1117, 1181HV Amsterdam, The Netherlands; Nutrition and Dietetics, Department of Internal Medicine, VU University Medical Center, De Boelelaan 1117, 1181HV Amsterdam, The Netherlands

## Abstract

**Introduction:**

Measurement of energy expenditure (EE) is recommended to guide nutrition in critically ill patients. Availability of a gold standard indirect calorimetry is limited, and continuous measurement is unfeasible. Equations used to predict EE are inaccurate. The purpose of this study was to provide proof of concept that EE can be accurately assessed on the basis of ventilator-derived carbon dioxide production (VCO_2_) and to determine whether this method is more accurate than frequently used predictive equations.

**Methods:**

In 84 mechanically ventilated critically ill patients, we performed 24-h indirect calorimetry to obtain a gold standard EE. Simultaneously, we collected 24-h ventilator-derived VCO_2_, extracted the respiratory quotient of the administered nutrition, and calculated EE with a rewritten Weir formula. Bias, precision, and accuracy and inaccuracy rates were determined and compared with four predictive equations: the Harris–Benedict, Faisy, and Penn State University equations and the European Society for Clinical Nutrition and Metabolism (ESPEN) guideline equation of 25 kcal/kg/day.

**Results:**

Mean 24-h indirect calorimetry EE was 1823 ± 408 kcal. EE from ventilator-derived VCO_2_ was accurate (bias +141 ± 153 kcal/24 h; 7.7 % of gold standard) and more precise than the predictive equations (limits of agreement −166 to +447 kcal/24 h). The 10 % and 15 % accuracy rates were 61 % and 76 %, respectively, which were significantly higher than those of the Harris–Benedict, Faisy, and ESPEN guideline equations. Large errors of more than 30 % inaccuracy did not occur with EE derived from ventilator-derived VCO_2_. This 30 % inaccuracy rate was significantly lower than that of the predictive equations.

**Conclusions:**

In critically ill mechanically ventilated patients, assessment of EE based on ventilator-derived VCO_2_ is accurate and more precise than frequently used predictive equations. It allows for continuous monitoring and is the best alternative to indirect calorimetry.

## Introduction

The optimal energy target in the first days of critical illness remains controversial [1–3]. Nonetheless, measurement of energy expenditure (EE) is important to prevent early overfeeding and later underfeeding, as both are associated with increased mortality [4–6]. EE can be accurately assessed with indirect calorimetry, which measures oxygen consumption (VO_2_) and carbon dioxide production (VCO_2_) from respiratory gases [7, 8]. EE is then calculated using the abbreviated formula published by Weir [9]:

$$ \mathrm{E}\mathrm{E}\ \mathrm{kcal}/\mathrm{day} = 3.941\times \mathrm{V}{\mathrm{O}}_2\left(\mathrm{L}/ \min \right)+1.11\times \mathrm{V}\mathrm{C}{\mathrm{O}}_2\left(\mathrm{L}/ \min \right)\times 1440 $$

Indirect calorimetry is often not available and is resource- and time-consuming. Daily assessment of EE is not feasible but could be important because EE is known to vary widely over time as a result of changing metabolic rate [10–12]. In the absence of indirect calorimetry, numerous predictive equations are used to estimate EE, including the Harris–Benedict equation and the European Society for Clinical Nutrition and Metabolism (ESPEN) guideline equation of 25 kcal/kg/day [13, 14]. These equations are notoriously inaccurate for individual critically ill patients, owing to large disease-, treatment-, and interindividual-related differences in metabolic rate [15–17]. The Penn State University and Faisy equations were especially developed for mechanically ventilated critically ill patients and include temperature and minute ventilation in the calculation of EE [15, 18]. The Penn State University equation is recommended by the Academy of Nutrition and Dietetics when indirect calorimetry is not feasible [19]. Validation studies for both equations are limited.

An alternative method to assess EE in mechanically ventilated critically ill patients could be the use of VCO_2_ measurements only. This is practical, as most mechanical ventilators provide the option to measure VCO_2_ continuously. When VCO_2_ is known, the Weir formula can be used to calculate VO_2_, assuming the respiratory quotient (RQ), which is the ratio between VCO_2_ and VO_2_. Its physiologic range of 0.67–1.2 depends on the type of the actually metabolized substrate provided that ventilation and acid–base balance are stable [20]. Although the latter vary during critical illness, in prolonged measurement periods metabolic CO_2_ production equals its excretion. Given these limitations, we hypothesized that EE could be assessed on the basis of ventilator-derived VCO_2_ using RQ of the administered nutrition and the rewritten Weir formula.

The aim of this study was to provide proof of concept that EE can be accurately assessed on the basis of ventilator-derived VCO_2_ and nutritional RQ and to determine whether this method is more accurate than frequently used predictive equations.

## Material and methods

### Study design and setting

This prospective observational study was conducted in the mixed medical-surgical adult intensive care unit (ICU) of the VU University Medical Center in Amsterdam, The Netherlands. The study was approved by the Medical Ethics Committee of the VU University Medical Center. The need for written informed consent was waived because indirect calorimetry is part of routine care in our ICU and imposes no burden on patients.

### Subjects

Inclusion criteria were age 18 years or older, mechanical ventilation, ICU stay of 3 days or more, and enteral or parenteral nutrition reaching at least two-thirds of calculated nutritional target. According to the standard practice of the unit, the initial nutritional target was an energy delivery as calculated with the revised Harris–Benedict equation [21], adding 20 % for stress and 10 % for activity [22, 23] and protein delivery of 1.2–1.5 g/kg preadmission body weight per day [24]. This target was adjusted based on indirect calorimetry measurements. An algorithm was used to determine the optimal nutritional product and amount needed to meet both protein and energy requirements [25]. Patients were excluded if they failed to meet accuracy criteria or safety criteria for indirect calorimetry, being a fraction of inspired oxygen (FiO_2_) greater than 0.6, air leakage through cuff or chest tubes, or a positive end-expiratory pressure (PEEP) greater than 14 cmH_2_O (arbitrary limit).

Our patient data management system (PDMS) (MetaVision; *i*MD*soft*, Düsseldorf, Germany) was used to routinely record demographic and clinical data; Acute Physiology and Chronic Health Evaluation (APACHE) II and III scores and APACHE IV predicted mortality [26–28]; diagnosis group; type, amount, and composition of feeding; and ventilation characteristics. Sedation was assessed by using the Ramsay Sedation Scale [29].

### Study protocol

Patient weight and height were recorded upon ICU admission. Preadmission weight and height were obtained, and, if not available, they were measured or estimated by a clinician. Indirect calorimetry was performed for 24 h. Simultaneously, 24-h minute-by-minute ventilator-derived VCO_2_, which is routinely exported to the PDMS, was recorded. After the first hour of measurement, type and amount of nutrition were adjusted to meet EE as measured with indirect calorimetry. All macronutrient intake during the study period, including propofol and glucose infusions, were routinely stored in the PDMS.

### Methods used to assess energy expenditure

#### Energy expenditure from indirect calorimetry

Twenty-four–hour indirect calorimetry was performed with a Deltatrac II MBM-200 Metabolic Monitor (Datex, Helsinki, Finland) connected to the ventilator. Before this study, an alcohol-burning test was performed to calibrate the metabolic monitor. Before each 24-h measurement, the metabolic monitor was prepared and calibrated according to the manufacturer’s instructions. Artifact suppression was turned on. For each patient, VCO_2_, VO_2_, RQ, and energy expenditure from indirect calorimetry (EE:Calorimetry) were recorded minute by minute and exported to a computer. For comparison, the mean 24-h value was calculated for each patient.

#### Energy expenditure from ventilator-derived volume of carbon dioxide and nutritional respiratory quotient

We use SERVO-i mechanical ventilators (Maquet, Rastatt, Germany) in our ICU. These have mainstream CO_2_ sensors connected to the airway adapter that measure end-tidal CO_2_. Sensors were calibrated before every study period and subsequently at 8-h intervals or more often if necessary. The SERVO-i ventilator calculates VCO_2_ from the product of CO_2_ concentration in expiratory air and the expiratory volume (VCO_2_ = volume × fraction of expired CO_2_).

VCO_2_ is displayed breath by breath and exported to the PDMS once each minute. For each patient, 24-h minute-by-minute VCO_2_ values were collected. To calculate energy expenditure from ventilator-derived volume of carbon dioxide and nutritional respiratory quotient (EE:VCO_2_), the average 24-h VCO_2_ (ml/min) was used.

Nutritional RQ was calculated considering 24-h macronutrient delivery, including calories provided by propofol (1.1 kcal/ml) and glucose (4 kcal/g). We assumed RQs of 1 for carbohydrates, 0.7 for fat, and 0.8 for protein. Nutritional RQ was calculated from the weighted average RQ for intake during the study period. For example, if the composition of the enteral formula was 16 % protein, 49 % carbohydrates, and 35 % fat, the nutritional RQ was calculated as 0.16 × 0.8 + 0.49 × 1 + 0.35 × 0.7 = 0.86.

After calculating nutritional RQ for each patient, EE:VCO_2_ was subsequently calculated using the following rewritten Weir formula:

$$ EE=3.941\times \mathrm{V}\mathrm{C}{\mathrm{O}}_2\left(\mathrm{L}/ \min \right)\div \mathrm{Nutritional}\ \mathrm{R}\mathrm{Q}+1.11\times \mathrm{V}\mathrm{C}{\mathrm{O}}_2\left(\mathrm{L}/ \min \right)\times 1440 $$

#### Energy expenditure derived from predictive equations

EE was calculated using four predictive equations: the Harris–Benedict equation [21], the ESPEN guideline equation [14], the Penn State University 2003b equation [15], and the Faisy equation [18].

Energy expenditure was calculated with the Harris–Benedict equation (EE:HB) as follows:

$$ \begin{array}{l}\mathrm{men}:88.362+13.397\times \mathrm{weight}\left(\mathrm{kg}\right)+4.799\times \mathrm{height}\left(\mathrm{cm}\right)-5.677\times \mathrm{age}\left(\mathrm{y}\right)\hfill \\ {}\mathrm{women}:447.593+9.247\times \mathrm{weight}\left(\mathrm{kg}\right)+3.098\times \mathrm{height}\left(\mathrm{cm}\right)-4.33\times \mathrm{age}\left(\mathrm{y}\right)\hfill \end{array} $$

Energy expenditure was calculated with the European Society for Clinical Nutrition and Metabolism guideline of 25 kcal/kg/day (EE:Esp25).

Energy expenditure was calculated with the Penn State University 2003b equation (EE:PSU) as follows:

$$ \mathrm{Mifflin}-\mathrm{St}\kern0.24em \mathrm{Jeor}\times 0.96+\mathrm{T} \max \times 167+\mathrm{V}\mathrm{e}\times 31\kern0.24em \hbox{-} \kern0.24em 6212 $$

The Mifflin-St Jeor calculation is as follows [30]:

$$ \begin{array}{l}\mathrm{men}:10\times \mathrm{weight}\left(\mathrm{kg}\right)+6.25\times \mathrm{height}\left(\mathrm{cm}\right)-5\times \mathrm{age}\left(\mathrm{y}\right)+5\hfill \\ {}\mathrm{women}:10\times \mathrm{weight}\left(\mathrm{kg}\right)+6.25\times \mathrm{height}\left(\mathrm{cm}\right)-5\times \mathrm{age}\left(\mathrm{y}\right)-161\hfill \end{array} $$

Tmax is highest body temperature during the 24-h study period, and Ve is mean minute ventilation during the 24-h study period.

Energy expenditure was calculated with the Faisy equation (EE:Faisy) as follows:

$$ 8\times \mathrm{weight}\;\left(\mathrm{kg}\right)+14\times \mathrm{height}\left(\mathrm{cm}\right)+32\times \mathrm{V}\mathrm{e}\left(\mathrm{L}/ \min \right)+94\times \mathrm{T}\hbox{-} 4834 $$

Ve is mean minute ventilation during the 24-h study period, and T is mean temperature during the 24-h study period.

### Endpoints

The primary endpoint was accuracy of EE:VCO_2_ using EE:Calorimetry as a gold standard. Secondary endpoints were the accuracy of EE:HB, EE:Esp25, EE:Faisy, and EE:PSU.

### Data analysis

Descriptive data are reported as mean [standard deviation (SD)], median (25th–75th percentile), or number (percentage) as appropriate. Student’s *t* test was used for comparison of paired data. Correlations were calculated using Pearson’s test, and strength of correlation was expressed as *r*. The accuracy of the different measurement methods was assessed in accordance with the ISO 5725 standard [31], which describes how accuracy can be defined in terms of bias and precision. Bias is the systematic error as compared with the gold standard (in this case EE:Calorimetry), whereas precision is the random (non-systematic) error of individual measurements. The inaccuracy of a measurement method can thus be due to a large bias (the systematic component), low precision (the random component), or both. Bias was calculated as the mean difference of EE:VCO_2_ (or equation-based EEs) and gold standard EE (EE:Calorimetry). EE was considered unbiased if the bias was less than 10 % of the gold standard EE [32]. Precision was quantified as the SD of the bias and the limits of agreement (2 SD). SDs of the different methods were compared using Levene’s test for equality of variances. Bland–Altman plots were used to graphically represent bias and limits of agreement [33]. Accuracy was further quantified by *accuracy rates*, which we defined as the proportion of patients for which the EE:VCO_2_ (or equation-based EE) predicted EE within 10 % and 15 % of gold standard EE:Calorimetry. We calculated greater than 25 % and greater than 30 % inaccuracy rates to quantify the occurrence of large errors, as the proportion of patients for which the EE:VCO_2_ (or equation-based EE) differed by more than 25 % or more than 30 % from gold standard EE:Calorimetry.

In a post hoc analysis, we calculated for which stress and activity factor the bias of the Harris–Benedict equation was lowest and used this equation in further data analysis (EE:HB15).

IBM SPSS 20 software (IBM, Armonk, NY, USA) was used for statistical analysis. A *p* value less than 0.05 was considered statistically significant.

## Results

**Fig. 1:**
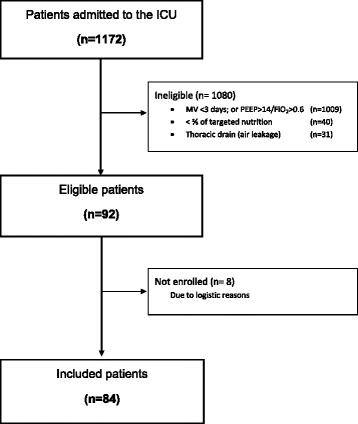
Consolidated Standards of Reporting Trials diagram representing the inclusion of patients. *FiO*_*2*_ fraction of inspired oxygen, *ICU* intensive care unit, *MV* mechanical ventilation, *PEEP* positive end-expiratory pressure

During the study period (20 March to 5 December 2013), 1172 patients were admitted to our ICU. Among them, 163 (13.9 %) were mechanically ventilated for more than 3 days with FiO_2_ 60 % or less and PEEP 14 cmH_2_O or less. Among these 163 patients, 123 (75 %) received about two-thirds of their nutritional energy target (defined by Harris–Benedict +30 %) and 92 of those 123 had no thoracic drains. Of the 92 eligible patients, 84 patients (91 %) were included (see Fig. 1). The main reason for missed inclusion was absence of a researcher. The included patients’ demographic, clinical, and nutritional characteristics are shown in Table 1. Twenty-six patients (31 %) were female. The most prevalent ICU admission diagnoses were post–cardiac arrest, postsurgery, and trauma. Twelve patients (14 %) had sepsis. The mean APACHE II score was 23.9 ± 8.4. Most patients were on pressure support ventilation (82 %). The mean total macronutrient intake during the 24-h study period was 1835 ± 627 kcal, including caloric intake from glucose and propofol infusions.

**Table 1:** Demographic, clinical, and nutritional characteristics of the study population

Characteristics	Data
Number of patients	84
Male, n (%)	58 (69)
Female, n (%)	26 (31)
Age, yr (mean ± SD)	63.5 ± 14.9
Height, cm (mean ± SD)	173.7 ± 7.8
Weight, kg (mean ± SD)	79.1 ± 16.0
BMI, kg/m^2^ (mean ± SD)	22.7 ± 4.4
APACHE II score (mean ± SD)	23.9 ± 8.4
APACHE III score (mean ± SD)	91.0 ± 34.3
APACHE IV predicted mortality (mean ± SD)	0.47 ± 0.31
ICU admission diagnosis, n (%)	
Trauma	15 (17.9)
Sepsis	12 (14.3)
Respiratory insufficiency	10 (11.9)
Postsurgery	18 (21.4)
Neurologic	4 (4.8)
Post–cardiac arrest	21 (25)
Cardiovascular	4 (4.8)
Length of ICU stay at time of study, days, median (IQR)	4.0 (3–6)
Ramsay Sedation Scale score,^a^ median (IQR)	5 (4–6)
Body temperature, °C (mean ± SD)	36.8 ± 0.8
Heart rate, beats/minute (mean ± SD)	91 ± 20
MAP, mmHg (mean ± SD)	84 ± 14
Norepinephrine, n (%)	33 (39.3)
CVVH, n (%)	8 (9.5)
Respiratory rate, breaths/min, median (IQR)	19 (15–24)
Minute volume, L/min (mean ± SD)	9.3 ± 3.2
Tidal volume, ml (mean ± SD)	462 ± 121
PaO_2_/FiO_2_ ratio, median (IQR)	220 (180–263)
PEEP, cmH_2_O, median (IQR)	8 (5–10)
Mechanical ventilation mode, n (%)	
PS/CPAP	69 (81.4)
PC	15 (17.9)
Type of nutrition, n (%)	
Enteral	73 (86.9)
Parenteral	4 (4.8)
Combination enteral and parenteral	7 (8.3)
Total nutritional intake, kcal/24 h (mean ± SD)	1748 ± 621
Total macronutrient intake,^b^ kcal/24 h (mean ± SD)	1835 ± 627
Length of mechanical ventilation, days, median (IQR)	8 (6–15)
Length of stay ICU, days, median (IQR)	11 (7–18)
Length of stay hospital, days, median (IQR)	23 (13–45)
ICU mortality, n (%)	29 (30.9)
Hospital mortality, n (%)	36 (38.3)

*APACHE* acute physiology and chronic health evaluation, *BMI* body mass index, *CPAP* continuous positive airway pressure, *CVVH* continuous venovenous hemofiltration, *ICU* intensive care unit *IQR* interquartile range, *MAP* mean arterial pressure, *PaO*_*2*_ */FiO*_*2*_ ratio of partial pressure of arterial oxygen to fraction of inspired oxygen, *PC* pressure control, *PEEP* positive end-expiratory pressure, *PS* pressure support, *SD* standard deviation

^a^Ramsay Sedation Scale scoring system: 1 = patient anxious and agitated or restless, or both; 2 = patient cooperative, orientated, and tranquil; 3 = patient responds to commands only; 4 = brisk response to a light glabellar tap or auditory stimulus; 5 = sluggish response to light glabellar tap or auditory stimulus; 6 = no response to the stimuli mentioned for scores 4 and 5

^b^Including intake from intravenous propofol and glucose

### Energy expenditure, VO_2_, VCO_2_, and RQ

Mean 24-h results for EE, VO_2_, VCO_2_, and RQ are presented in Table 2. Mean 24-h EE:Calorimetry was 1823 ± 408 kcal. Mean 24-h EE:VCO_2_ was 1963 ± 431 kcal, which was significantly higher than EE:Calorimetry (*p* < 0.001) (see Table 2).

**Table 2:** Mean 24-h results of VCO_2_, VO_2_, RQ, and EE measurements

	Mean ± SD	*p* value (vs. calorimetry)
VCO_2_ (ml/min)		
Calorimetry	225 ± 47	
Ventilator	240 ± 52	<0.001
VO_2_ (ml/min)		
Calorimetry	265 ± 59	
RQ		
Calorimetry	0.8592 ± 0.0473	
Nutrition	0.8636 ± 0.0119	0.410
Nutrition^a^	0.8629 ± 0.0151	0.485
Energy expenditure (kcal/24 h)		
Calorimetry	1823 ± 408	
VCO_2_-derived	1963 ± 431	<0.001
HB equation	1576 ± 257	<0.001
Esp25	1979 ± 400	<0.001
Faisy equation	1999 ± 269	<0.001
PSU	1801 ± 314	0.431
HB15	1813 ± 295	0.724

*Calorimetry* measured with indirect calorimetry, *Esp25* European Society for Clinical Nutrition and Metabolism -guideline equation of 25 kcal/kg/day, *HB15* Harris–Benedict equation with 15 % added; *PSU* Penn State University 2003b equation, *RQ* respiratory quotient, *SD* standard deviation, *VCO*_*2*_ *-derived* carbon dioxide production, *VCO*_*2*_ *-derived* from ventilator-derived carbon dioxide production and nutritional respiratory quotient, *VO*_*2*_ oxygen consumption

^a^Including macronutrient intake from intravenous propofol and glucose

### Correlation

**Fig. 2:**
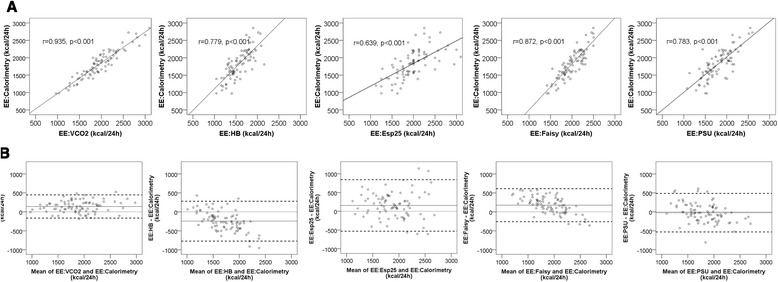
Correlation and agreement between the methods used to assess energy expenditure (EE) and gold standard indirect calorimetry. **a** Regression plots showing the correlation between the different methods used to assess EE and gold standard indirect calorimetry. **b** Bland–Altman plots showing the agreement between the methods used to assess EE and gold standard indirect calorimetry. The *solid lines* indicate the bias (mean difference with indirect calorimetry). The *thick dashed lines* indicate the limits of agreement (bias ±2 standard deviations). Every dot represents 1 of 84 patients. The *x*-axis represents the mean of the method used to assess EE and gold standard indirect calorimetry. The *y*-axis represents the difference in EE in kilocalories per 24 h between the method used and gold standard indirect calorimetry. EE:Esp25, Energy expenditure calculated with the European Society for Clinical Nutrition and Metabolism guideline equation of 25 kcal/kg/day; EE:Faisy, Energy expenditure calculated with the Faisy equation; EE:HB, Energy expenditure calculated with the Harris–Benedict equation; EE:PSU, Energy expenditure calculated with the Penn State University 2003b equation; EE:VCO_2_, Energy expenditure from ventilator-derived volume of carbon dioxide and nutritional respiratory quotient

EE:VCO_2_ correlated strongly with EE:Calorimetry (r = 0.935). The equation-based EEs correlated less strongly and the correlation coefficient was lowest for EE:Esp25 (r = 0.639) (see Fig. 2).

### Bias (mean difference of EE:VCO_2_ and predictive equations with EE:Calorimetry)

Bland–Altman plots are shown in Fig. 2. The bias of EE:VCO_2_ was +141 ± 153 kcal/24 h (7.7 % of EE:Calorimetry). This was significantly lower than the bias of EE:HB (−246 ± 263 kcal/24 h, *p* < 0.001), comparable to the bias of EE:Faisy (+176 ± 218 kcal/24 h, *p* = 0.226) and EE:Esp25 (+156 ± 344 kcal/24 h, *p* = 0.709), but higher than the bias of EE:PSU (−22 ± 254 kcal/24 h, *p* < 0.001). In post hoc analysis, we calculated that the bias of the Harris–Benedict equation was lowest with a stress and activity factor of +15 % (−10 ± 257 kcal/24 h). See Table 3 for detailed results. The bias of ventilator-derived VCO_2_ was 14.7 ml/min (6.5 % of VCO_2_:Calorimetry). The bias of nutritional RQ was 0.0037 (0.4 % of RQ:Calorimetry).

**Table 3:** Accuracy of the methods used to assess EE, expressed as bias, precision, and accuracy and inaccuracy rates

	Bias		Precision		Accuracy quantified			
	Mean difference in kcal/day, 95% CI	Mean difference (% of gold standard EE)	SD of bias (Levene’s *F*-test^a^)	Bland–Altman limits of agreement	Accuracy rates		Inaccuracy rates	
					<10 %	<15 %	>25 %	>30 %
Method								
EE:VCO_2_^b^	+141, +107 to +174	7.7 %	153	−166 to +447	61 %	79 %	2 %	0 %
EE:HB^c^	+246	13.5 %	263	−722 to +280	31 %	52 %	13 %	5 %
	−303 to −189	*p* < 0.001	(*F* 14.1)		*p* = 0.001	*p* < 0.001	*p* < 0.002	*p* < 0.01
			*p* < 0.001					
EE:Esp25^d^	+156,	8.6 %	344	−531 to +843	40 %	56 %	25 %	14 %
	+81 to +230	*p* = 0.709	(*F* 31.1)		*p* = 0.009	*p* = 0.002	*p* < 0.001	*p* < 0.001
			*p* < 0.001					
EE:Faisy^e^	+176,	9.7 %	218	−260 to + 612	45 %	61 %	17 %	12 %
	+129 to +233	*p* = 0.226	(*F* 9.0)		*p* = 0.01	*p* = 0.003	*p* < 0.001	*p* < 0.001
			*p* = 0.003					
EE:PSU^f^	−22,	1.2 %	254	−529 to +458	54 %	75 %	10 %	6 %
	−77 to +33	*p* < 0.001	(*F* 11.9)		*p* = 0.341	*p* = 0.582	*p* = 0.05	*p* = 0.023
			*p* < 0.001					
Post hoc calculation								
EE:HB15^g^	−10	0.5 %	257	−524 to +504	55 %	71 %	10 %	6 %
	−66 to +46	*p* < 0.001	(*F* 12.3)		*p* = 0.435	*p* = 0.285	*p* = 0.05	*p* = 0.023
			*p* < 0.001					

*EE* energy expenditure, *EE:Esp25* energy expenditure calculated with the European Society for Clinical Nutrition and Metabolism guideline equation of 25 kcal/kg/day, *EE:Faisy* energy expenditure calculated with the Faisy equation, *EE:HB15* energy expenditure calculated with the Harris–Benedict equation with 15 % added, *EE:PSU* energy expenditure calculated with the Penn State University 2003b equation, *EE:VCO*_*2*_ energy expenditure from ventilator-derived volume of carbon dioxide and nutritional respiratory quotient, *HB* Harris–Benedict equation

Less than 10 % and less than 15 % accuracy rates represent the proportion of patients for which EE:VCO_2_ (or equation-based EE) predicted EE within 10 % and within 15 %, respectively, of gold standard EE:Calorimetry. Greater than 25 % and greater than 30 % inaccuracy rates represent the proportion of patients for whom EE:VCO_2_ (or equation-based EE) differed by more than 25 % and more than 30 %, respectively, from gold standard EE:Calorimetry

All *p* values are relative to EE:VCO_2_.

*F*-test and *p* value reflect the comparison of the variance of the mean difference of EE:VCO_2_ and EE from equations. The higher the *F*-value, the higher the difference of the variances. *p* < 0.05 indicates that the variance of the mean difference is significantly different from EE:VCO_2_.

^a^Levene’s *F*-test on similar variances

Bias: ^b^ vs. ^c^ *p* < 0.001; ^b^ vs. ^d^ *p* = 0.709; ^b^ vs. ^f^ *p* = <0.001; ^b^ vs. ^e^ *p* < 0.001; ^b^ vs. ^g^ *p* = 0.226; ^c^ vs. ^d^ *p* < 0.001; ^c^ vs. ^f^ *p* < 0.001; ^c^ vs. ^e^ *p* < 0.001; ^c^ vs. ^g^ *p* < 0.001; ^d^ vs. ^f^ *p* = 0.001; ^d^ vs. ^e^ *p* < 0.001; ^d^ vs. ^g^ *p* = 0.652; ^f^ vs. ^e^ *p* = 0.762; ^e^ vs. ^g^ *p* < 0.001

### Precision

**Fig. 3:**
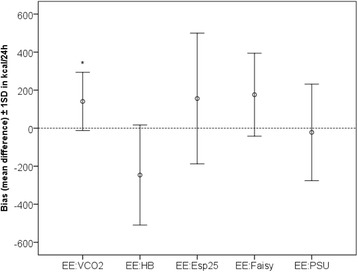
Bias and precision of the methods used to assess energy expenditure (EE). The *x*-axis shows the different methods used to assess EE. The *y*-axis represents the bias (mean difference with gold standard indirect calorimetry) and the precision (±1 standard deviation) in kilocalories per day. *Variance of the bias significantly smaller than that of the predictive equations. EE:Esp25, Energy expenditure calculated with the European Society for Clinical Nutrition and Metabolism guideline equation of 25 kcal/kg/day; EE:Faisy, Energy expenditure calculated with the Faisy equation; EE:HB, Energy expenditure calculated with the Harris–Benedict equation; EE:PSU, Energy expenditure calculated with the Penn State University 2003b equation; EE:VCO_2_, Energy expenditure from ventilator-derived volume of carbon dioxide and nutritional respiratory quotient

Limits of agreement were smallest for EE:VCO_2_ (−166 to +447 kcal/24 h) The SD of the bias of EE:VCO_2_ was significantly smaller than that of all equation-based EE values (see Table 3 and Figs. 2 and 3).

### Accuracy and inaccuracy rates

**Fig. 4:**
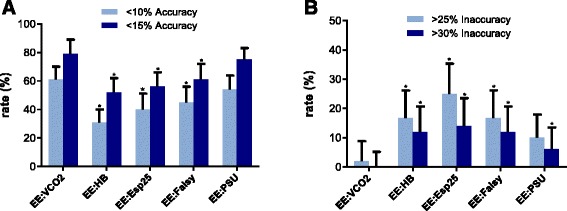
Accuracy and inaccuracy of the different methods quantified in less than 10 % and less than 15 % accuracy rates and greater than 25 % and greater than 30 % inaccuracy rates. **a** Less than 10 % and less than 15 % accuracy rates were defined as the proportion of patients for whom energy expenditure (EE) was predicted within 10 % and within 15 % of gold standard EE:Calorimetry. **b** Greater than 25 % and greater than 30 % inaccuracy rates were defined as the proportion of patients for whom EE differed by more than 25 % and more than 30 % from gold standard EE:Calorimetry. The *x*-axis shows the different methods used to assess EE. The *y*-axis represents the accuracy rates or inaccuracy rates in percentages. The *error bars* reflect upper bounds of 95 % confidence intervals. *Significantly different from EE:VCO_2_ (*p* values are shown in Table 3). EE:Esp25, Energy expenditure calculated with the European Society for Clinical Nutrition and Metabolism guideline equation of 25 kcal/kg/day; EE:Faisy, Energy expenditure calculated with the Faisy equation; EE:HB, Energy expenditure calculated with the Harris–Benedict equation; EE:PSU, Energy expenditure calculated with the Penn State University 2003b equation; EE:VCO_2_, Energy expenditure from ventilator-derived volume of carbon dioxide and nutritional respiratory quotient

Less than 10 % and less than 15 % accuracy rates of EE:VCO_2_ were 61 % and 79 %, respectively. These were significantly higher than those of EE:HB, EE:Esp25, and EE:Faisy but not significantly different from EE:PSU and EE:HB15. Less than 25 % and less than 30 % inaccuracy rates of EE:VCO_2_ were 2 % and 0 %, respectively. The less than 30 % inaccuracy rate of EE:VCO_2_ was significantly lower than that of all equation-based EE values (Table 3 and Fig. 4).

## Discussion

The present prospective observational study in critically ill mechanically ventilated patients provides proof of concept that EE can be accurately calculated from EE:VCO_2_. Furthermore, it shows that this method is more precise than frequently used predictive equations. The bias or systematic error of EE:VCO_2_ was 141 kcal/24 h, indicating that EE:VCO_2_ as derived from the ventilator systematically overestimates EE compared with gold standard EE:Calorimetry. However, this bias corresponds to a relative error of only 7.7 % of the gold standard, whereas up to 10 % is considered acceptable according to a consensus statement [32]. The precision or random error component of EE:VCO_2_, expressed as the SD of the bias and compared between methods by using Levene’s test, is visualized by the width of the limits of agreement in the Bland–Altman plots and in Fig. 2. The precision of EE:VCO_2_ was significantly better than that of the equations.

The accuracy rates of EE:VCO_2_ were higher than those of all predictive equations, but not significantly so for EE:PSU. However, the inaccuracy of EE:PSU was higher, with greater than 25 % and greater than 30 % inaccuracy rates of 10 % and 6 %, respectively, indicating that in more than half of the patients with inaccuracy of greater than 25 %, the error was even larger—namely, more than 30 % difference from EE as measured by indirect calorimetry.

High inaccuracy rates were found for EE:HB and EE:Esp25, making these equations unacceptable for use in critically ill patients. In all, EE:VCO_2_ appears to be the most precise equation and EE:PSU and EE:HB15 the most unbiased equations. Despite a better estimation of the mean EE of the study population, the inferior precision of EE:PSU and EE:HB15 led to higher inaccuracy rates, which may result in severe over- or underfeeding in a considerable number of patients. Thus, for the individual patient, EE:VCO_2_ performs best.

We further explored the source of the bias of EE:VCO_2_, which can be due to inaccuracy of the VCO_2_ measurement or the RQ estimation. We found an unexpected bias of ventilator-derived VCO_2_ of 14.7 ml/min (6.5 % of VCO_2_:Calorimetry). Assuming an RQ of 0.86, which is the RQ of most nutritional products, this systematic error accounts for 120 kcal/24 h (i.e., 85 % of the bias of EE:VCO_2_). We noted the largest differences between ventilator-derived and calorimetry-derived VCO_2_ in patients with extreme variations in respiratory rate and tidal volume. Rapid and irregular breathing may lead to errors in ventilator-derived VCO_2_ due to dyssynchrony between the flow and the CO_2_ measurement. Furthermore, the ventilator exports a single-breath VCO_2_ value once each minute to the PDMS, which can lead to high variability in patients with irregular breathing. One way of improving the accuracy of the EE:VCO_2_ method is the development of more accurate VCO_2_ analyzers in mechanical ventilators, such as by more frequent sampling and data export.

A second source of error and an important limitation of our study is that the actual RQ of the patients was not known. In the present study, we used nutritional RQ. However, during critical illness, RQ is influenced not by actual nutritional intake alone. An unknown and uninhibitable part of energy is derived from endogenous sources, and there are different illness-related degrees of protein synthesis or catabolism, lipogenesis or lipolysis, and gluconeogenesis or glycolysis. Because of the uncertainty of actual RQ when endogenous sources are used for energy, we could not correct RQ if nutritional intake was less than EE. However, our patients received more than two-thirds of actual EE, and this is the time point when measurement of EE becomes relevant. RQ is also influenced by periods of hypo- and hyperventilation (e.g., induced by stress or sedation or in respiratory compensation for metabolic acidosis or alkalosis). This will temporarily modulate VCO_2_ [34]; however, over 24 h, mean VCO_2_ reflects CO_2_ produced by metabolism. Although nutritional RQ did indeed not correlate with measured RQ:Calorimetry, only 15 % of the bias of EE:VCO_2_ was attributable to the difference between nutritional RQ and RQ:Calorimetry.

In our study, additional calories provided during the study period by glucose and propofol were taken into account. With a single exception in a patient who received large amounts of glucose 40 %, these additional calories did not substantially change nutritional RQ and subsequently EE:VCO_2_.

Mehta et al. tested the accuracy of a VCO_2_ based equation to calculate EE in critically ill children [35]. Metabolic data from mechanically ventilated children was used to derive this equation. The equation was then applied to a second dataset of critically ill children to test accuracy. They found superiority of the simplified equation over standard equations. These findings further strengthen the concept of using VCO_2_ measurement instead of estimating equations to calculate EE in critically ill adults and children. It should be noted, however, that the VCO_2_ in the Mehta study was not independently measured; it was derived from the metabolic monitor. Thus, a direct comparison between EE:Calorimetry and a ventilator-derived or separate module-derived EE:VCO_2_ was not performed. Mehta et al. mentioned this as a limitation of their study. Also, measurement periods were relatively short. We were able to perform simultaneous 24-h VCO_2_ and indirect calorimetric measurements in a large and representative population of ICU patients ventilated for more than 3 days, providing information on real-time total EE.

Indirect calorimetry remains the gold standard. However, the most validated system, the Deltatrac, is no longer being manufactured. While we await new, accurate, affordable metabolic monitors, EE:VCO_2_ could be of great benefit for ICUs that do not have indirect calorimetry available. The method can also be used to monitor fluctuations in EE over time and to identify patients at risk for being over- or underfed. EE:VCO_2_ allows for daily adjustment of nutrition in ventilated patients. This may be important because metabolic rate and associated energy requirements vary widely during the day and during the course of disease [11, 12, 36, 37]. Another major advantage of EE:VCO_2_ is that the calculation of EE is independent of body length and weight, thereby reducing error.

We are aware of the fact that not all ICUs have mechanical ventilators that measure VCO_2_ continuously. Most modern ventilators do have this option available and cost less than a metabolic monitor. Of note, the present validation was performed with one type of mechanical ventilator. We do not know the accuracy of VCO_2_ measurements with other ventilators.

We excluded patients with FiO_2_ exceeding 0.6 for reliability reasons and patients with PEEP above 14 cmH_2_O because of risks associated with disconnection when connecting the indirect calorimeter to the ventilator. Therefore, our method was not validated in this population. Nonetheless, we suppose that EE:VCO_2_ is reliable in all mechanically ventilated patients, regardless of ventilator settings, provided that air leakage is not present.

The most important message of this study is that EE (kcal/day) can be calculated at the bedside as 8.19 × VCO_2_ (ml/min). This equation is derived from the rewritten Weir formula using an RQ of 0.86, which is the RQ of most nutritional products, and after converting liters per minute to milliliters per minute.

## Conclusions

In critically ill mechanically ventilated adult patients, the assessment of EE from ventilator-derived VCO_2_ is accurate and more precise than frequently used predictive equations. It allows for continuous monitoring and provides the best alternative to gold standard indirect calorimetry. Future studies are necessary to improve accuracy of the VCO_2_ measurement, to detect sources of error, and to investigate whether daily adjustment of nutrition based on ventilator-derived EE improves the outcome of ICU patients.

## Key messages

- EE from ventilator-derived VCO_2_ is accurate and more precise than predictive equations.
- This method allows for continuous monitoring and is the best alternative to indirect calorimetry.
- EE (kcal/day) can be calculated at the bedside as 8.19 × VCO_2_ (ml/min).

## Abbreviations

APACHE: Acute Physiology and Chronic Health Evaluation
BMI: Body mass index
CPAP: Continuous positive airway pressure
CVVH: continuous venovenous hemofiltration, EE, Energy expenditure
EE:Esp25: Energy expenditure calculated with the European Society for Clinical Nutrition and Metabolism guideline equation of 25 kcal/kg/day
EE:Faisy: Energy expenditure calculated with the Faisy equation
EE:HB: Energy expenditure calculated with the Harris–Benedict equation
EE:PSU: Energy expenditure calculated with the Penn State University 2003b equation
EE:VCO_2_: Energy expenditure from ventilator-derived volume of carbon dioxide and nutritional respiratory quotient
ESPEN: European Society for Clinical Nutrition and Metabolism
FiO_2_: Fraction of inspired oxygen
ICU: Intensive care unit
IQR: Interquartile range
MAP: mean arterial pressure
PaO_2_: Partial pressure of arterial oxygen
PC: Pressure control
PDMS: Patient data management system
PEEP: Positive end-expiratory pressure
PS: Pressure support
RQ: Respiratory quotient
SD: Standard deviation
T: Mean temperature during the 24-h study period
Tmax: Maximum body temperature during the 24-h study period
VCO_2_: carbon dioxide production
Ve: Mean minute ventilation during the 24-h study period
VO_2_: oxygen consumption
